# Recurrence of lumbar disk herniation after microdiscectomy: a two-center retrospective analysis of 1214 cases and identification of risk factors

**DOI:** 10.55730/1300-0144.5691

**Published:** 2023-05-09

**Authors:** Bülent GÜLENSOY, Ebru GÜZEL, Müge KUZU KUMCU, Hüseyin KARASU, Serkan ŞİMŞEK, Aslan GÜZEL

**Affiliations:** 1 Department of Neurosurgery, Lokman Hekim University, Ankara, Turkiye; 2 Department of Radiology, Medical Point Hospital, Gaziantep, Turkiye; 3 Department of Neurology, Lokman Hekim University, Ankara, Turkiye; 4 Department of Neurosurgery, Medical Point Hospital, Gaziantep, Turkiye

**Keywords:** Recurrent lumbar disc herniation, microdiscectomy, risk factors, the Pfirrmann grading system

## Abstract

**Background/aim:**

To present the incidence of recurrent lumbar disc herniation (RLDH) and to identify radiological and patient-related risk factors that lead to recurrence after lumbar disc herniation (LDH) treatment with microdiscectomy.

**Materials and methods:**

Between January 2013 and December 2021, 1214 patients who had undergone microdiscectomy for LDH were included in this retrospective study. Patients were divided into two groups, the recurrent group and the non-recurrent group, and their demographic, clinical and radiologic characteristics were recorded. The association between the variables and RLDH was assessed by univariate and multivariable logistic regression analyses.

**Results:**

Mean ages were similar in the recurrent (51.48 ± 13.63) and non-recurrent(50.38 ± 14.53) groups (p=0.232). Males represented 59.6% of the recurrent group and 49.8% of the non-recurrent group (p=0.002). Multivariable logistic regression revealed that being a male (p=0.009), diabetes mellitus (p=0.038), smoking (p<0.001), grade 4&5 disc degeneration (p<0.001), and having protruded (p=0.002), extruded LDH (p<0.001), paracentral (p=0.008) and foraminal LDH (p=0.008) were independently associated with recurrence.

**Conclusion:**

To reduce RLDH frequency and need for revision surgery, modifiable risk factors should be minimized before and after the initial surgery. Also, in patients with unmodifiable risk factors, patients should be clearly informed about the risk for recurrence and possible alternative treatment methods should be considered.

## 1. Introduction

Lumbar disc herniation (LDH) is the most common cause of low back pain, leg pain and work loss related with them [1,2]. While 90% of patients with LDH can be treated conservatively, 10% require surgical treatment [3,4]. Compared to conservative treatment, surgery allows faster and more effective pain relief, and prevents progression of motor deficits due to LDH [5]. However, recurrence and reoperation are among the common outcomes of surgery, and complications such as such as hematoma, infection, dural injury and nerve root injury have been reported [4,6,7].

Despite advances in surgical techniques, postoperative LDH recurrence continues to demonstrate a high rate, ranging from 0.5% to 25% [4,6]. Recurrent LDH (RLDH)increases risks for debilitating pain, disability, resurgery and additional burden on the healthcare system [8]. Unfortunately, the rate of reoperation due to RLDH varies between 3% and 11% and there are studies showing that revision spine surgeries have poor outcomes and a higher complication likelihood compared to primary surgery [2,9,10].

Because of its high rate and the mentioned adversities that arise from it, many researchers have turned to investigating risk factors that lead to RLDH in order to reduce its incidence. The most commonly reported ones may classified as follows: Patient-related factors: Age [11,12], sex [13,14,15], body-mass index (BMI) [16,17], diabetes mellitus(DM) [5,16], smoking [2,18] and hereditary factors such as biomechanical [3] and anatomical differences (lumbosacral transitional vertebra [4] and intervertebral discspace[5,16]); Pre-surgical radiological risk factors: Modic changes [5,16], Pfirrmann grade [19,20], type [18,21] and location [22,23] of disc herniation [20]; Surgical riskfactors: Surgeon experience [22] and type of primary surgery [21,24]; Post-surgical riskfactors: High-intensity postoperative activity [13,25] and occupation [18]. Although there are many studies on the subject, most of the related studies have either a small number of participants [16,19,20] or a small number of parameters investigated [2,24], or both[1,7,13,18].

In this study, we aimed to present the incidence of RLDH and to determine radiological and patient-related risk factors that are associated with post-surgical RLDH in patients who underwent microdiscectomy for LDH, with the inclusion of a large number of parameters and a large number of participants.

## 2. Patients and methods

### 2.1 Study design and ethical issues

This retrospective study was carried out by inclusion of patients from two Neurosurgery departments, Lokman Hekim University Hospital, Ankara, Turkey and Medical ParkGaziantep Hospital, Gaziantep, Turkey. Ethical approval for this study was provided by the Research Ethics Committee of the Faculty of Medicine of Lokman Hekim University(Date: 29.03.2022 number: 2022-58). It was carried out in accordance with the ethical standards set forth in the 1964 Declaration of Helsinki and its amendments.

### 2.2. Study population and data collection

A total of 1214 patients who underwent open-approach microdiscectomy for LDH in the two hospitals, from January 2013 to December 2021, were included in the study. Among these, 327 (26.9%) had developed RLDH (recurrent group), while the rest did not have recurrence (non-recurrent). To be eligible for inclusion in the study, patients had to meet the following criteria: 1) be between 18 and 70 years old, 2) have recurrent low back pain with radiculopathy at least 6 months after primary lumbar disc surgery, 3) have recurrent radicular pain unresponsive to conservative treatment for at least 6 weeks, 4) have recurrent low back pain with progressive neurological deficits 6 months after surgery, and 5) have magnetic resonance imaging of the lumbosacral spine showing disc herniation at the same level as the primary discectomy. Patients who developed RLDH within the first 6 months after the index surgery, those whose symptoms persisted after surgery, those with serious surgical or post-surgical complications, patients treated through endoscopicdiscectomy, patients with RLDH at a different level/side compared to before the index surgery, those with a history of LDH surgery at another center before the index operation, cases with less than 12 months follow-up period after index surgery, those with spinalinstability, and patients with low back pain without leg pain were excluded from the study. All demographic data including age and sex, anthropometric data including BMI (kg/m^2^), clinical data including comorbidities, smoking status, and all disease-related data determined by magnetic resonance (MR) imaging before the initial surgery [including level, side (right or left), type and location of disc herniation and degree of disc degeneration (Pfirrmann grade)] [26] were obtained.

All relevant data and recurrence-related features were retrospectively recorded via use of the hospitals’ computer databases. Recorded MR images were evaluated by an experienced radiologist. Patients whose recent information could not be reached from hospital records or those who did not attend follow-up were contacted by phone to obtain information about their recent status. In order to determine RLDH in these contacted patients (some of which stated that they undergone re-operation in other hospitals), the hospitals were called by phone and 94 necessary information was obtained from the operating physician. Participants who could not be reached were excluded from the study.

### 2.3. Definitions and tools

#### 2.3.1 Recurrent lumbar disc herniation

Recurrent lumbar disc herniation was defined as confirmation of disc herniation at the same level and side as the primary LDH by MR imaging, after more than 6 months of pain-free and untreated interval from the index operation [27].

#### 2.3.2 The Pfirrmann grading system

Lumbar disc degeneration classification was performed by experienced radiologists using T2-weighted MR images according to The Pfirrmann grading system (PGS) (26). PGS is a system that divides the extend of lumbar disc degeneration into 5 grades according to these 4 features: Intervertebral disc structure, distinction of the nucleus and the annulus, signal intensity of intervertebral discs, and height of intervertebral discs (26). Due to the heterogeneous distribution in the number of patients in terms of Pfirrmann grade, patients with grade 4&5 and patients with grade 1&2&3 were pooled to create two groups in the regression model.

#### 2.3.3 Type and location of disc herniation

The type of disc herniation was determined by distinguishing between protrusion, extrusion, and sequestration using T1- and T2-weighted MR images. Protruded type herniation is defined as disc herniation in which the ‘neck’ is wider than the herniated fragment. Extruded type herniation is defined as disc herniation in which the ‘neck’ is narrower than the herniated fragment. Sequestered type herniation describes a fragment that is no longer contiguous with the disc space [14,28,29]. The location of the disc herniation was classified using T1- and T2-weighted MR images as central, paracentral, foraminal, and far lateral (30).

### 2.4. Statistical Analysis

Two-tailed p-values of less than 0.05 were considered statistically significant. All analyses were performed on IBM SPSS Statistics for Windows, Version 25.0 (IBM Corp., Armonk, NY, USA). For the normality check, histograms and Q-Q plots were used. Data are given as mean ± standard deviation for continuous variables (with respect to normality of distribution results), and frequency (percentage) are used for categorical variables. Age was analyzed with the independent samples t test. Categorical variables were analyzed with chi-square tests (Pearson, Yate’s correction) or the Fisher’s exact test. Odds ratios(OR) for recurrence were calculated by using univariable logistic regression, and those with statistical significance were included in the multivariable logistic regression model to determine independent risk factors associated with recurrence.

## 3. Results

The mean age of the recurrent group was 51.48 ± 13.63 years, which was similar to the mean age of the non-recurrent group (50.38 ± 14.53) (p = 0.232). 59.6% of recurrent group, and 49.8% of non-recurrent group were male, and this difference in sex distribution was significant (p = 0.002). According to the results of the univariate analysis, the percentage of patients with BMI of >30 (p = 0.004), DM (p = 0.001), LDH on the left(p = 0.049), grade 4 disc degeneration according to PGS (p<0.001), protruded type discherniation (p<0.001) and who are smokers (p = 0.004) were significantly higher in the recurrent group. The percentage of patients with grade 2 and grade 3 disc degeneration(p<0.001), sequestered type disc herniation (p<0.001), and central and far lateral 140 localization of disc herniation (p = 0.003) were significantly higher in the non recurrent group **(**Table 1).

Multivariable logistic regression revealed that sex, DM, smoking, PGS grade, type of discherniation and location of disc herniation were independent risk factors associated with RLDH. Male patients had 1.474-fold higher risk of RLDH than female patients (OR:1.474, 95% CI: 1.101 – 1.973, p = 0.009). Patients with DM had 1.654-fold higher risk of RLDH than those without (OR: 1.654, 95% CI: 1.028 – 2.663, p = 0.038). Smokers had 147 2.023-fold higher risk of recurrence than non-smokers (OR: 2.023, 95% CI: 1.455 -2.812, p < 0.001). Patients with grade 4&5 disc degeneration had 4.651-fold higher risk of RLDH than patients with grade 1&2&3 disc degeneration (OR: 4.651, 95% CI: 3.375 -6.409, p < 0.001). Patients with protruded type LDH had 2.324-fold higher risk of recurrence than patients with sequestered type LDH (OR: 2.324, 95% CI: 1.377 – 3.921, p = 0.002) and patients with extruded type LDH had 2.516-fold higher risk of recurrence than patients with sequestered LDH (OR: 2.516, 95% CI: 1.621 – 3.906, p < 0.001). Patients with paracentral herniation had 5.271-fold higher risk of RLDH than patients with central LDH (OR: 5.271, 95% CI: 1.550 – 17.926, p = 0.008) and patients with foraminal LDH had 6.460-fold higher risk of RLDH than patients with central LDH (OR: 6.460, 95% CI: 1.643 – 25.398, p = 0.008) (Table 2).

## 4. Discussion

Our study identified several pre-surgical risk factors that are independently associated with the development of RLDH, including male sex, diabetes mellitus, smoking, Pfirrmann grade 4&5 disc degeneration, protruded and extruded type LDH, and paracentral and foraminal localization. It is important to avoid surgery beyond the first due to elevated risks for surgical complications, and therefore, identifying and recognizing risk factors associated with recurrence is crucial [7]. In addition, the rate of RLDH in this study was found to be 26.9%, which is higher than those reported in the literature [4,6]. Recurrent post-microdiscectomy LDH is a common occurrence, with reported rates ranging from 0.5% to 25% [4,6].

Various pre-operative radiological parameters and classifications have been investigated for their role in predicting RLDH [5,18,19,22]. The severity of pre-surgery discdegeneration is among the most frequently investigated factors. Pfirrmann et al. proposeda classification showing the degree of disc degeneration based on the reflection of the structural changes in disc and loss of disc height on T2-weighted MR imaging as a result of intervertebral disc degeneration [26]. We found that the risk of RLDH was higher inpatients with Pfirrmann grade 4&5. Similarly, in the univariate analysis of a retrospective study, higher Pfirrmann grade was found to be significantly associated with increased rate of RLDH and revision surgery [19]. Kim et al. showed that the risk of RLDH was greater in patients with moderate (i.e. grades 3, 4, 5, and 6) disc degeneration compared to other grades of the Modified PGS [14]. The study of Dora et al. concluded that low-grade discdegeneration (grades 1–3) is an important risk factor for RLDH [31], which is supported by another recent study [28]. It was stated that the outcomes of these three studies are explained by the theory put forth by Kirkaldy-Willis and Farfan. This theory suggests that disc degeneration progresses at a natural cycle including temporary dysfunction, instability and re-stabilization [14,28,32]. Of note, there are also researchers who argue that the degree of disc degeneration is not a predictor for RLDH [1,16,33]. Normally, it is considered that disc degeneration impairs the post-discectomy healing process and efficient reconstruction of the external annulus suggesting that the risk of RLDH will increase as disc degeneration increases [14]. However, these inconsistencies in the results necessitate the existence of more comprehensive studies to show the definitive effect of disc degeneration. Another radiological criterion that has been frequently discussed in studies is LDH type. In the present study, we found that the risk of recurrence was higher in protruded and extruded LDHs compared to sequestered LDH. In one study, it was reported that the recurrence rate after open discectomy was significantly higher in the protruded type than in the sequestered and extruded type [34].

Another study concluded that a contained disc protrusion is almost three times more likely to require revision surgery than extruded or sequestered discs [35]. Huang et al.’s meta-analysis also obtained consistent results [36].

On the other hand, in a recent study, extruded and sequestered disc herniation were identified as risk factors for recurrence [25], which is supported by other studies [15,37]. Whereas, many other studies argue that herniation type has no effect on RLDH [1,16,28].

Researchers who found the risk of RLDH to be higher in protruded type hernias claimed that this might be due to the difficulty of complete removal of the herniated disc in protruded hernia [34]. Some researchers who thought that extruded and sequestered hernias increase the risk of RLDH attributed this to the avascular structure of the intervertebral disc, the absence of healing of the annulus fibrosis, and development of re-hernia from the defects formed at the site after the initial surgery [15].

According to another view, extruded and sequestered disc herniation cannot be completely removed and residual nucleus pulposus fragments can lead to postoperative RLDH [25]. As can be seen, the relationship between LDH type and RLDH is still unclear. The last radiological criterion we investigated was hernia localization, and we found that paracentral and foraminal disc herniation may be a risk factor for RLDH compared to central and far lateral localization. Yao et al. argued that the probability of RLDH after treatment of central herniation with percutaneous endoscopic lumbar discectomy (PELD)was higher compared to paramedian herniation. However, it was emphasized that there as on for this result was probably the difficulty in removing the central hernia with the surgical technique applied [22]. Another study of patients with initial PELD showed that patients with paracentral disc herniation were more likely to experience early recurrence compared to patients with central and distant lateral hernias [23]. The authors of this study also suggested that when the working channel is placed with a more horizontal trajectory for central disc herniation and with a more vertical trajectory for paracentral and far lateral disc herniation, the remaining disc material can be minimized, thus reducing the possibility of recurrence [23]. However, it is notable that the number of studies that did not find a relationship between hernia localization and hernia recurrence is not low[8,11,20,30].

The most important common feature of these three radiological parameters is that there are a large number of studies whose results are contradictory. Therefore, in such a heterogeneous pool of results, we think it would be more accurate to investigate why the results are so different. The effect of these parameters on the risk of RLDH seems to be highly likely to be affected by the type of surgery performed, the individual biomechanical and anatomical differences of the patients included in the study, and the limited understanding regarding the pathophysiological mechanism between these factors and LDH [15, 34, 36]. A more reliable risk classification can be made after these causes of variation can be adjusted for, controlled or stratified.

LDH is a multifactorial disease in which biomechanical, anatomical, hereditary, clinical and environmental factors play a role [4,16]. Therefore, many other factors other than radiological parameters are likely to affect the risk of recurrence. In this study, we identified male sex, presence of DM and preoperative smoking as predictors of RLDH in addition to the radiological parameters discussed above. There are several studies with conflicting results regarding sex distribution. Some report RLDH is higher in men[13,14], some in women [15,33], while various others find similar frequencies [1,2,16].

Higher exposure of men to physical stress factors that can strain the intervertebral disc, such as sports or heavy lifting, may explain why this rate is higher in men in some studies[14]. On the other hand, the opinion that it is seen more in women suggests an association with the presence of higher BMI among women [15]. Our multivariable analyses showed that recurrence was independently associated with being male. With regard to DM, there are studies showing increased RLDH risk among these patients [5,16], while others have not found any relationship [1,19]. DM may facilitate the formation of both primary LDH and RLDH with possible mechanisms such as reducing the proteogly can and glycosaminoglycan density in the disc and/or disrupting nutrition and healing of the disc[20,36]. Similarly, according to some researchers, smoking increases the risk of RLDH[2,5,13,18], while others have not found significant relationships [1,33]. The effects of smoking include disruption of the nutrition and oxygenation of the disc, inhibition of cell proliferation, collagen synthesis, extracellular matrix synthesis, and increased intra-discpressure [2,16].

Although this study explored a large set of parameters among a high number of patients, we could not evaluate a few parameters which have been identified as risk factors for RLDH in the literature [22,28]. For example, the diversity of surgeons performing the operation and surgery type are important factors that may affect the success of the operation, and therefore, the risk of recurrence [3,5]. Because LDH is a multifactorial disease, it is an expected result that the results of studies investigating risk factors for recurrence are different. Another important issue is that there is no generally accepted definition of RLDH. We see that many different definitions of RLDH have been made in many studies [1,3,7,18,33]. This may contribute to the inconsistencies. Conducting comprehensive prospective studies including all possible and conflicting factors, identifying definitive risk factors for RLDH, and elucidating pathophysiological mechanisms with respect to these factors can be useful approached for future studies.

## 5. Study limitations

While evaluating the results of this study, it is necessary to consider potential pitfalls. Although this is a two-center study with a large number of participants, thereby enabling reliable generalizability, it should be noted that data were recorded retrospectively. Other risk factors such as occupation [18], high-intensity postoperative activity [23,25] and other radiological parameters [28] could not be investigated. As the follow-up period of each patient was not the same and only symptomatic patients who applied to the health institution for possible recurrence were included in the RLDH group, the actual incidence of RLDH may not have been obtained. In addition, revision surgery-related data and the exact time interval between recurrence and initial surgery were not available for all patients in the recurrent group; thus, analyses concerning these parameters were not included in the study. The impact of patients’ lifestyle differences and occupational intensity on recurrence was not investigated. Also, The difference in complication rates between the surgeons could not be evaluated since some of the patients included in the study had their first operation performed at a different center.

## 6. Conclusion

In conclusion, male sex, DM, smoking, Pfirrmann grade 4&5 disc degeneration, protruded and extruded type LDH, and paracentral and foraminal localization were found to be independent pre-surgical risk factors associated with RLDH development. In order to reduce the rate of RLDH and revision surgery, it is critical to minimize the effects of modifiable risk factors (both before and after the initial surgery). Also, patients with unmodifiable risk factors must be made aware of the high likelihood of recurrence after surgery, and alternative treatment methods should be offered when possible. However, first of all, a common definition of RLDH should be made and more comprehensive, multicenter studies should be conducted to clarify the conflicting results regarding recurrence-related risk factors.

## Figures and Tables

**Table 1:** Summary of patient and herniation characteristics with regard to recurrence

		Recurrence	
	Total (n=1214)	Absent (n=887)	Present (n=327)
**Age**	50.68 ± 14.29	50.38 ± 14.53	51.48 ± 13.63
**Sex**			
Female	577 (47.5%)	445 (50.2%)	132 (40.4%)
Male	637 (52.5%)	442 (49.8%)	195 (59.6%)
**Body mass index**			
≤30	651 (53.6%)	498 (56.1%)	153 (46.8%)
>30	563 (46.4%)	389 (43.9%)	174(53.2%)
**Diabetes mellitus**	92 (7.6%)	53 (6.0%)	39 (11.9%)
**Hypertension**	152 (12.5%)	107 (12.1%)	45 (13.8%)
**Coagulopathy**	12 (1.0%)	8 (0.9%)	4 (1.2%)
**Smoking**	373 (30.7%)	252 (28.4%)	121 (37.0%)
**Level of disc herniation**			
L1–L2	16 (1.3%)	12 (1.4%)	4 (1.2%)
L2–L3	49 (4.0%)	36 (4.1%)	13 (4.0%)
L3–L4	145 (11.9%)	112 (12.6%)	33 (10.1%)
L4–L5	642 (52.9%)	452 (51.0%)	190 (58.1%)
L5-S1	362 (29.8%)	275 (31.0%)	87 (26.6%)
**Side of disc herniation**			
Right	561 (46.2%)	425 (47.9%)	136 (41.6%)
Left	653 (53.8%)	462 (52.1%)	191 (58.4%)
**Pfirrmann grading system**			
Grade 1	0 (0.0%)	0 (0.0%)	0 (0.0%)
Grade 2	37 (3.0%)	37 (4.2%)	0 (0.0%)
Grade 3	553 (45.6%)	465 (52.4%)	88 (26.9%)
Grade 4	610 (50.2%)	375 (42.3%)	235 (71.9%)
Grade 5	14 (1.2%)	10 (1.1%)	4 (1.2%)
**Type of disc herniation**			
Protruded	209 (17.2%)	138 (15.6%)	71 (21.7%)
Extruded	816 (67.2%)	591 (66.6%)	225 (68.8%)
Sequestered	189 (15.6%)	158 (17.8%)	31 (9.5%)
**Location of disc herniation**			
Central	35 (2.9%)	32 (3.6%)	3 (0.9%)
Paracentral	1081 (89.0%)	782 (88.2%)	299 (91.4%)
Foraminal	55(4.5%)	35(3.9%)	20 (6.1%)
Far lateral	43 (3.5%)	38 (4.3%)	5 (1.5%)

Data are given as mean ± standard deviation for continuous variables according to normality of distribution and as frequency (percentage) for categorical variables

**Table 2:** Risk Factors of Recurrence, Logistic Regression Analyses

	Univariable		Multivariable
	OR (95% CI)	p	OR (95% CI)
**Age**	1.005 (0.997 – 1.014)	0.232	
**Sex, Male**	1.487 (1.150 – 1.923)	**0.002**	1.474 (1.101 – 1.973)
**Body Mass Index, >30**	1.456 (1.129 – 1.878)	**0.004**	1.261 (0.955 – 1.664)
**Diabetes Mellitus**	2.131 (1.380 – 3.291)	**0.001**	1.654 (1.028 – 2.663)
**Hypertension**	1.163 (0.800 – 1.691)	0.428	
**Coagulopathy**	1.361 (0.407 – 4.549)	0.617	
**Smoking**	1.480 (1.132 – 1.935)	**0.004**	2.023 (1.455 – 2.812)
**Level of Disc Herniation**	0.981 (0.843 – 1.142)	0.808	
**Side of Disc Herniation, Left**	1.292 (1.000 – 1.669)	0.051	
**Pfirrmann Grading System, Grade 4&5**	3.541 (2.681 – 4.677)	**<0.001**	4.651 (3.375 – 6.409)
**Type of Disc Herniation** (1)			
Protruded	2.622 (1.623 – 4.237)	**<0.001**	2.324 (1.377 – 3.921)
Extruded	1.940 (1.282 – 2.937)	**0.002**	2.516 (1.621 – 3.906)
**Location of Disc Herniation** (2)			
Paracentral	4.078 (1.240 – 13.418)	**0.021**	5.271 (1.550 – 17.926)
Foraminal	6.095 (1.653 – 22.472)	**0.007**	6.460 (1.643 – 25.398)
Far lateral	1.404 (0.311 – 6.332)	0.659	1.346 (0.286 – 6.329)
**Nagelkerke R** **^2^**	-		0.187

OR: Odds ratio, CI: Confidence Interval.

(1) Reference Category: Sequestered.

(2) Reference Category: Central
